# Echocardiography and cardiovascular magnetic resonance based evaluation of myocardial strain and relationship with late gadolinium enhancement

**DOI:** 10.1186/s12968-019-0559-y

**Published:** 2019-08-08

**Authors:** Jennifer Erley, Davide Genovese, Natalie Tapaskar, Nazia Alvi, Nina Rashedi, Stephanie A. Besser, Keigo Kawaji, Neha Goyal, Sebastian Kelle, Roberto M. Lang, Victor Mor-Avi, Amit R. Patel

**Affiliations:** 1 Department of Internal Medicine / Cardiology, German Heart Center, Berlin, Germany; 2 Department of Medicine, University of Chicago Medical Center, 5758 S. Maryland Avenue, MC9067, Chicago, IL 60637 USA; 3 Department of Cardiac, Thoracic and Vascular Sciences, University of Padua, Padua, Italy; 4 Department of Biomedical Engineering, Illinois Institute of Technology, Chicago, IL USA; 5 Department of Internal Medicine/Cardiology, Charité Campus Virchow Klinikum, Berlin, Germany; 6 DZHK (German Center for Cardiovascular Research), Partner Site Berlin, Berlin, Germany; 7 Department of Cardiology, Riverside Medical Center, Kankakee, IL USA

**Keywords:** Cardiac imaging, Left ventricular function, Myocardial deformation, Myocardial scar

## Abstract

**Objectives:**

We sought to: (1) determine the agreement in cardiovascular magnetic resonance (CMR) and speckle tracking echocardiography (STE) derived strain measurements, (2) compare their reproducibility, (3) determine which approach is best related to CMR late gadolinium enhancement (LGE).

**Background:**

While STE-derived strain is routinely used to assess left ventricular (LV) function, CMR strain measurements are not yet standardized. Strain can be measured using dedicated pulse sequences (strain-encoding, SENC), or post-processing of cine images (feature tracking, FT). It is unclear whether these measurements are interchangeable, and whether strain can be used as an alternative to LGE.

**Methods:**

Fifty patients underwent 2D echocardiography and 1.5 T CMR. Global longitudinal strain (GLS) was measured by STE (Epsilon), FT (NeoSoft) and SENC (Myocardial Solutions) and circumferential strain (GCS) by FT and SENC.

**Results:**

GLS showed good inter-modality agreement (*r*-values: 0.71–0.75), small biases (< 1%) but considerable limits of agreement (− 7 to 8%). The agreement between the CMR techniques was better for GLS than GCS (r = 0.81 vs 0.67; smaller bias). Repeated measurements showed low intra- and inter-observer variability for both GLS and GCS (intraclass correlations 0.86–0.99; coefficients of variation 3–13%). LGE was present in 22 (44%) of patients. Both SENC- and FT-derived GLS and GCS were associated with LGE, while STE-GLS was not. Irrespective of CMR technique, this association was stronger for GCS (AUC 0.77–0.78) than GLS (AUC 0.67–0.72) and STE-GLS (AUC = 0.58).

**Conclusion:**

There is good inter-technique agreement in strain measurements, which were highly reproducible, irrespective of modality or analysis technique. GCS may better reflect the presence of underlying LGE than GLS.

## Introduction

Despite the important role left ventricular (LV) ejection fraction (EF) plays in clinical practice, it is influenced by heart rate and loading conditions. Given these limitations, there has been a considerable interest in myocardial strain as an alternative measure of myocardial performance. Depending on fiber direction in the different myocardial layers, longitudinal, circumferential and radial strain can be differentially impacted [1]. Studies have shown that myocardial strain is less dependent on loading conditions than EF [2, 3], and, as a result, better reflects subtle changes in the underlying myocardial substrate [4, 5]. Strain measurements using speckle tracking echocardiography (STE) have been widely reported, including evidence that strain is a better predictor of outcomes than EF [6]*.* Due to superior reproducibility of strain [7–9], it is recommended for clinical use to detect chemotherapy-related cardiotoxicity [10] and to evaluate cardiac involvement in infiltrative diseases, such as amyloidosis or sarcoidosis [11]. Several recent studies suggested STE strain as a potential surrogate for cardiovascular magnetic resonance (CMR) late gadolinium enhancement (LGE) imaging [9, 12], the current reference standard for detection of scar and infiltrative disease. This would be useful in cases where CMR is not available, gadolinium contrast is contraindicated, or in patients at greater risk of adverse long term events, such as children and pregnant women.

As CMR becomes more widely utilized, the need to incorporate strain assessment into the CMR exam is being increasingly recognized. Although several CMR techniques to assess strain have been recently described, this methodology is still not fully developed. One technique analyzes strain from cine-CMR images using feature tracking (FT) algorithms, similar to STE. FT-derived strain has been shown to detect ischemia during dobutamine stimulation [13] and to independently predict outcomes in patients with dilated cardiomyopathy [14]. Newer CMR techniques include dedicated pulse sequences that create images with strain information encoded into a color display to facilitate visual assessment of abnormalities. Although this strain-encoding (SENC) requires additional imaging, it does not significantly prolong the exam [15], and has higher spatial and temporal resolution even than myocardial tagging, the current reference standard for strain [16–18]*.* The ability of SENC to detect myocardial infarction and define its transmurality has been reported [1, 17]. While echocardiographic studies showed that global longitudinal strain (GLS) is superior to global circumferential strain (GCS) in its ability to detect subtle myocardial abnormalities due to better reproducibility [11, 19], this has not been confirmed for CMR-derived strain.

Despite the growing number of strain related studies in the literature, the methodology of CMR strain has not been standardized, and it is not known to what extent these measurements are interchangeable with each other and with STE. Also, the reproducibility of these techniques is not well established. Furthermore, it is not clear whether the relationship of strain measurements (by either STE or CMR) with LGE is strong enough for strain to be considered as a surrogate. Finally, the differences between the strain components in this context are not well established. Accordingly, we sought to: 1) determine the inter-technique agreement between STE, FT and SENC strain measurements, 2) compare their reproducibility, and 3) determine which modality and technique shows the strongest association with LGE.

## Methods

### Study population and design

We retrospectively studied 50 patients who underwent CMR imaging (including SENC and LGE) and transthoracic 2D echocardiography at the University of Chicago, Chicago, Illinois, USA over a one-year period. Patients under 18 years of age and those who underwent a cardiac intervention between the two imaging tests were excluded. No patients were excluded on the basis of image quality of either modality. The Institutional Review Board approved this retrospective study with a waiver of consent.

**Fig. 1:**
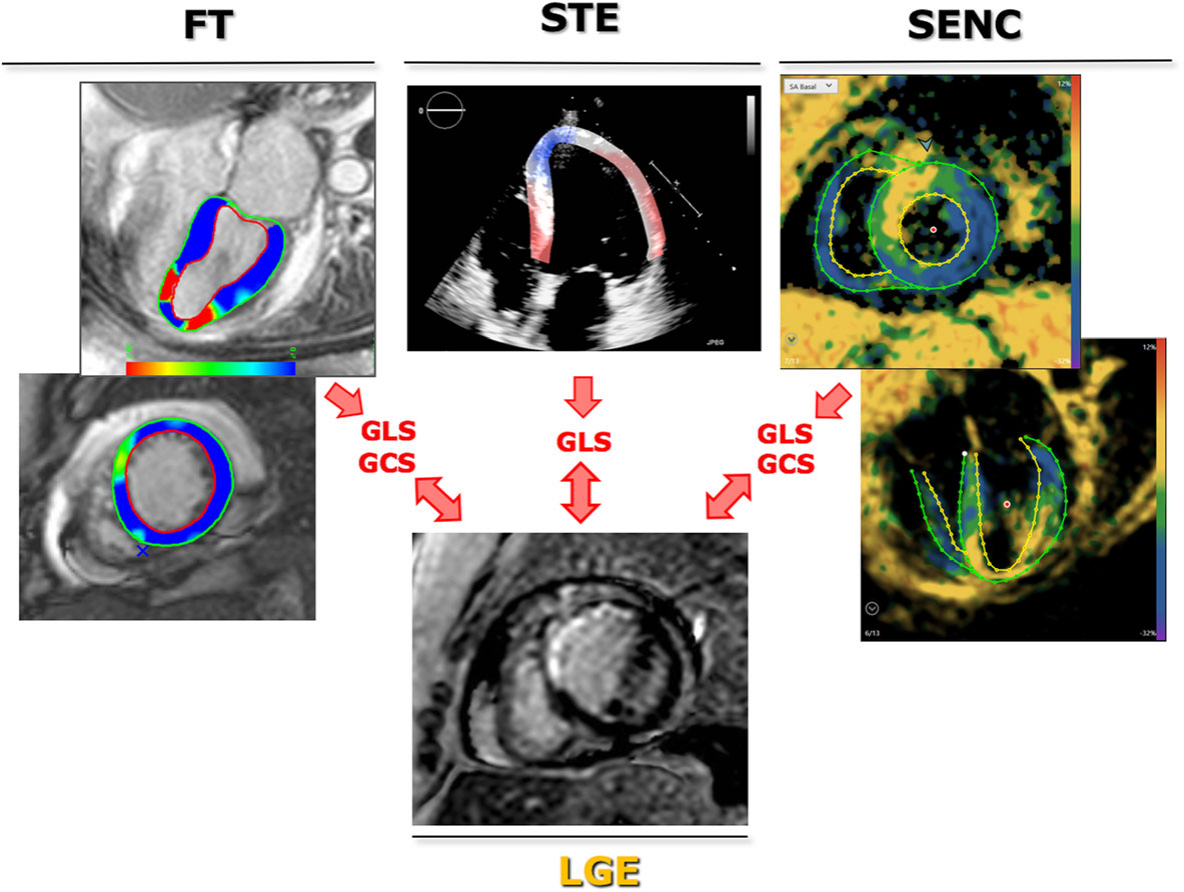
Schematic representation of the study design (see text details). Note: the images in the figure highlight the three techniques in a patient with a prior myocardial infarction in the territory of the left anterior descending coronary artery. The transmural late gadolinium enhancement in the mid-distal anterior, mid-distal anteroseptal and distal septum, suggesting lack of viability in these areas, is represented by different colors for the different techniques. FT, feature tracking; GCS, global circumferential strain; GLS, global longitudinal strain; LGE, late gadolinium enhancement; STE, speckle tracking echocardiography; SENC, strain encoding

Figure 1 schematically depicts the study design. The above three techniques were used to measure strain: feature tracking (FT) and strain encoding (SENC) images were analyzed to obtain both global longitudinal strain (GLS) and global circumferential strain (GCS), while speckle tracking echocardiography (STE) was used to obtain GLS. These measurements were compared between them and also correlated with presence of LGE.

### Echocardiographic imaging and analysis

Transthoracic echocardiographic imaging was performed using the iE33 system with the X5–1 probe (Philips Healthcare, Best, the Netherlands). Apical long-axis, LV-focused two-, three- and four-chamber (2Ch, 3Ch and 4Ch) views were acquired, after optimizing the sector size, gain, depth, compress and time-gain compensation. Frame rate was maximized (73 ± 20 fps) by increasing the depth and decreasing the sector width.

The images were stored digitally and measured offline according to the guidelines [20] by an experienced reader, blinded to clinical data and all prior strain measurements and LGE findings. End-diastole (ED) was identified as the frame coinciding with the peak of the QRS complex, whereas end-systole (ES) was identified as the frame with the smallest LV cavity. LV GLS analysis was performed on the three long-axis views using vendor independent speckle-tracking software (Echo Insight, Epsilon Imaging, Ann Arbor, Michigan, USA). This software is based on tracking ultrasound speckles frame-by-frame in order to quantify myocardial deformation. It has been previously validated by comparisons against other established techniques used to measure myocardial strain [3, 4, 7].

**Fig. 2:**
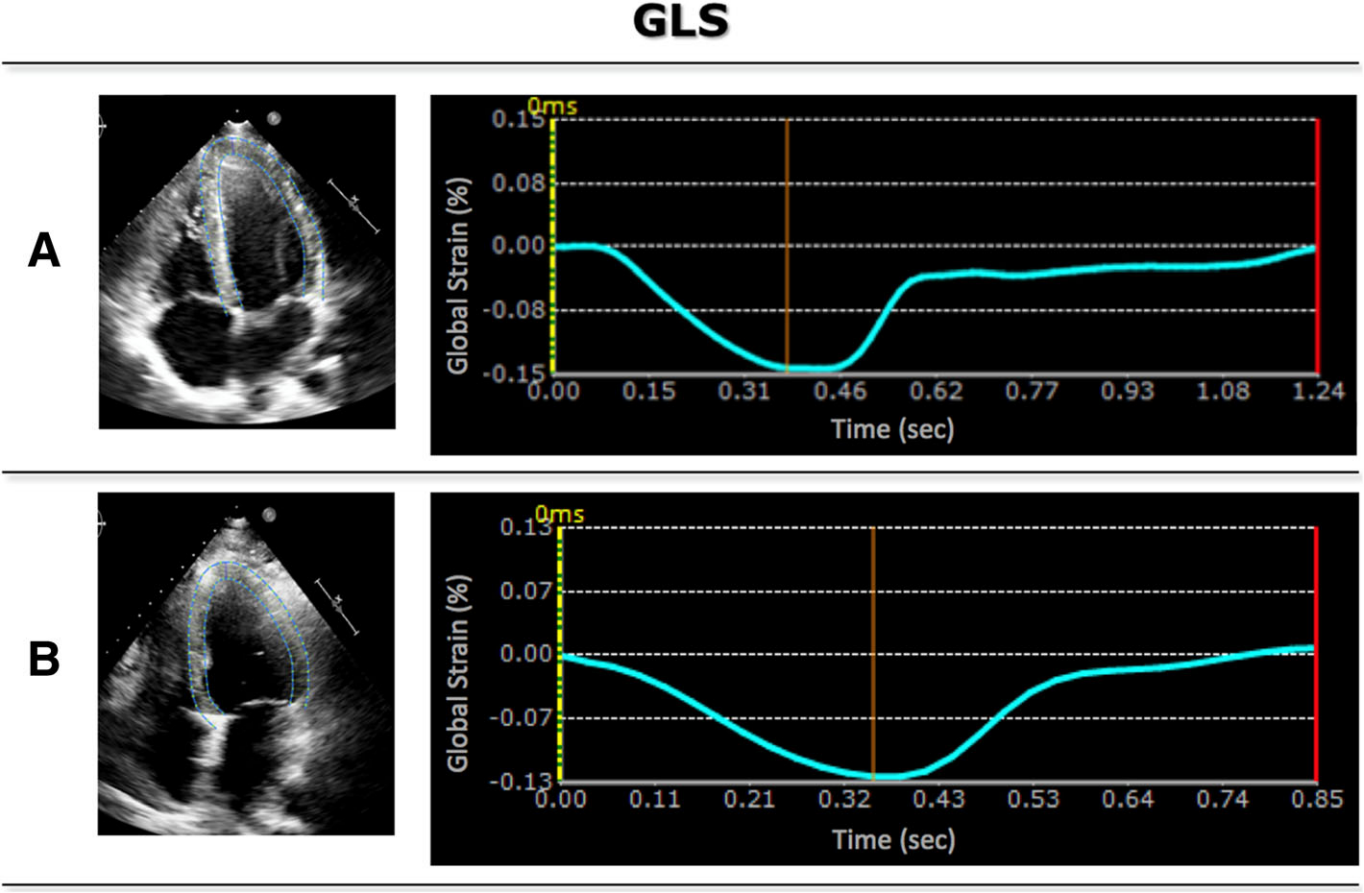
Example of STE (4Ch) images, showing tracing contours, and corresponding strain curves in a patient with no LGE (**a**) and a patient with cardiac manifestation of sarcoidosis (**b**)

Specifically, for each view, strain analysis was performed by manually tracing at ED the region of interest along the endocardial border from the mitral valve annulus to the LV apex and back to the annulus (Fig. 2a). The software then automatically tracked the endocardial contours throughout the cardiac cycle. Manual adjustments were made to the contours as needed to optimize tracking. All views were segmented according to the American Heart Association (AHA) guidelines and segmental strain was calculated automatically throughout the cardiac cycle. GLS was calculated throughout the cardiac cycle, resulting in a time-strain curve for each view (Fig. 2). Peak GLS values were averaged for the three views, resulting in a unique GLS value for each patient.

### Cine CMR imaging and feature tracking analysis

CMR images were acquired on a 1.5 T scanner using a 5-channel surface coil (Achieva, Philips Healthcare). Retrospectively gated cine images were acquired using a balanced steady-state free precession pulse sequence in the standard long-axis views (2Ch, 3Ch, 4Ch) and short-axis slices (6 mm thickness, 4 mm gap), covering the LV from base to apex. Scanning parameter were: TR = 2.9 ms, TE = 1.5 ms, flip angle 60°, temporal resolution 30-40 ms.

FT was performed offline by an experienced observer, blinded to all prior strain measurements and LGE findings, using vendor independent software (SuiteHEART, Neosoft, Pewaukee, Wisconson, USA). Similar to echocardiographic speckle tracking, the FT algorithm identifies image features in the myocardium that are consistently identifiable throughout the cardiac cycle, and tracks them frame-by-frame to quantify myocardial deformation. This is achieved by the following five steps: (1) deformation models are created based on b-splines using contours and images; (2) position images are created by calculating how much each pixel within the myocardium is displaced over the cardiac cycle; (3) strain tensor is calculated; (4) tensor image is transformed from Cartesian coordinates to the radial/cross-radial coordinates; and (5) velocity and strain rate tensors are calculated using the central difference.

**Fig. 3:**
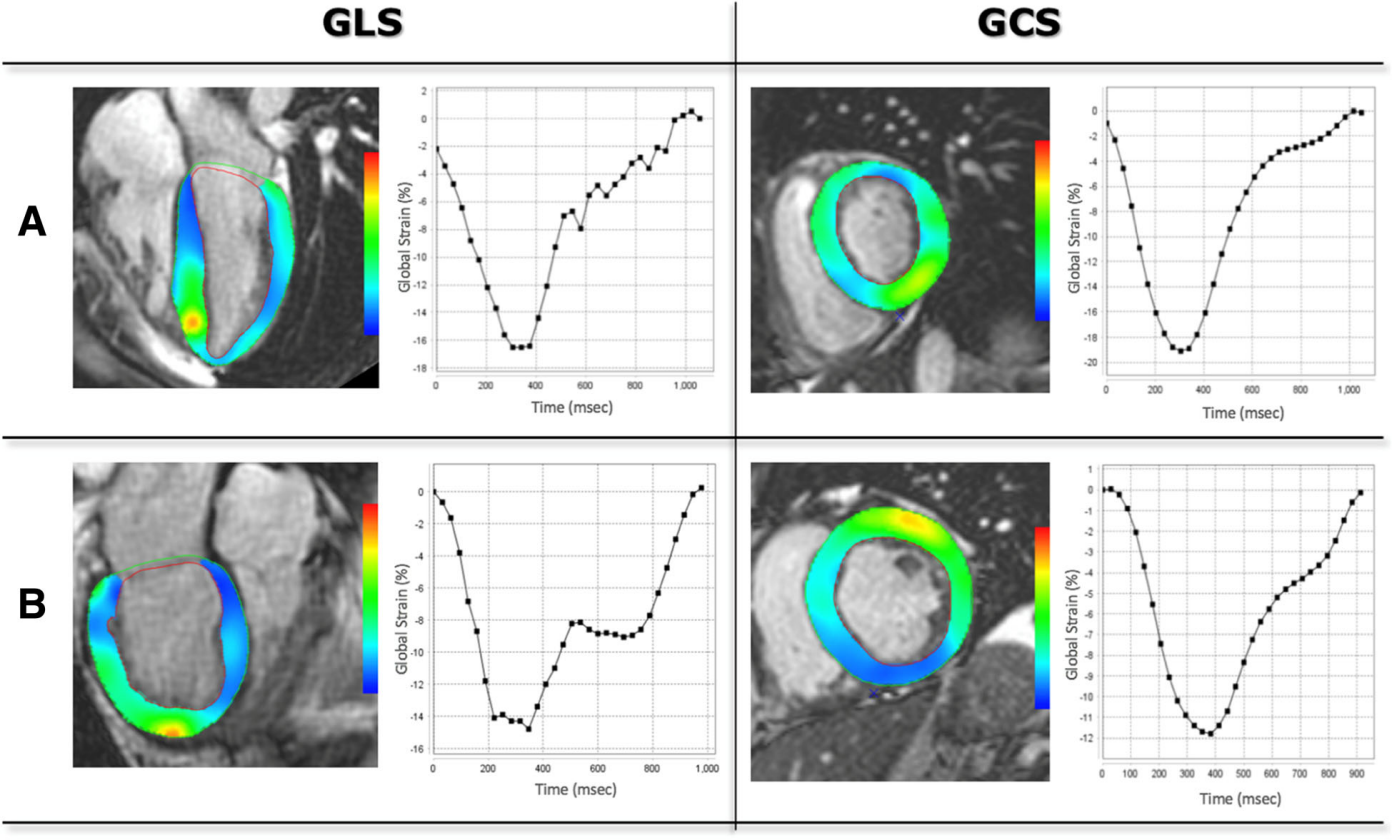
Example FT (4Ch, SAX Basal) images, showing tracing contours, and corresponding strain curves in the same two patients **a** and **b** as in Fig. 2

The long-axis cine images were used to determine GLS and short-axis slices covering the entire heart to determine GCS. First, ED and ES were determined automatically in each view and manually corrected as needed. The tracing of the region of interest in the long-axis images was performed by an automated machine-learning based process, tracking epi- and endocardial contours from the mitral valve annulus to the apex and back to the annulus. These contours were then reviewed on every frame throughout the cardiac cycle and manually corrected as needed to optimize endocardial detection and tracking while taking care to exclude papillary muscles and endocardial trabeculae from the LV cavity (Fig. 3a). All views were segmented according to the AHA guidelines and segmental strain was calculated automatically throughout the cardiac cycle. If segments were inadequately tracked, tracing was repeated until optimal tracking was achieved. Peak systolic GLS and GCS were calculated as a mean value of all segments (Fig. 3).

### CMR strain encoding and analysis

SENC images were acquired in three long-axis (2Ch, 3Ch, 4Ch) and three short-axis views (basal, mid, apical) with the following settings: TR = 13 ms; TE = 0.7 ms; FA = 30°; 256x256mm2; slice thickness = 10 mm; 24 ms SENC magnetization preparation prior to continuous acquisition of 40 ms (3 spiral interleaves) per temporal frame over 1 R-R cycle.

GLS was quantified using the three short-axis slices and GCS using the long-axis slices by the same observer three weeks after the analysis of the FT images to prevent bias, using vendor independent software (Myostrain 5.0, Myocardial Solutions, Morrisville, North Carolina, USA). Unlike STE and FT, SENC measures strain in the direction perpendicular to the imaging plane: circumferential from the long-axis and longitudinal from the short-axis images. Radial strain is not usually assessed using SENC. This is achieved by using specialized pulse sequences designed to measure strain and generate color-encoded strain maps superimposed on a static anatomic image of the heart [18].

**Fig. 4:**
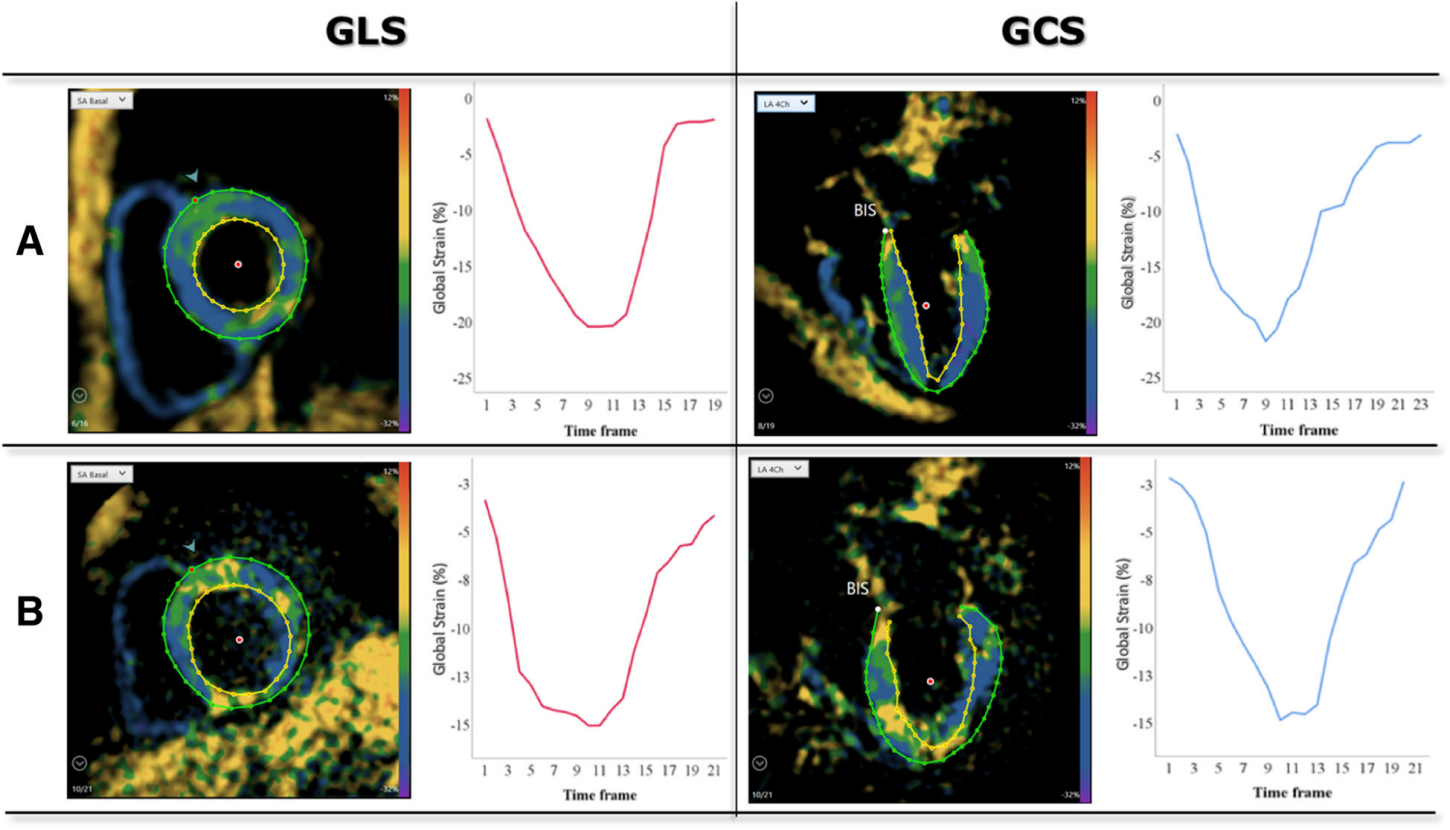
Example of SENC (4-Ch, basal short axis) images, showing tracing contours, and corresponding strain curves in the same two patients **a** and **b** as in Figs. 2 and 3

ED and end-systole (ES) were selected manually in all slices according to the size of the myocardial cavity and the color-coding of the images, representing the state of contraction (blue = contracting- yellow = relaxing). Epi- and endocardial contours were drawn manually at ES, again using the mitral valve annulus and apex as landmarks in the long-axis views and excluding the papillary muscles and trabeculae from the LV cavity (Fig. 4). GLS and GCS were automatically calculated for each view and then averaged. No segments were excluded from analysis.

### Late gadolinium enhancement

LGE images were acquired in the same long-axis planes as the cine images and also in the short-axis slices covering the entire LV, 5–10 min after the infusion of gadolinium contrast (OmniscanTM or MultiHance TM, 0.05–0.1 mmol/kg, injected at 4 ml/s, IV), using a T1-weighted gradient echo pulse sequence with a phase sensitive inversion recovery reconstruction (TR = 4.5 ms; TE = 2.2 ms; TI = 250–300 ms, flip angle 30°, flip angle 5°, voxel size = 2x2x10 mm, SENSE factor = 1–2, no gaps). An inversion time scout sequence was used to select an inversion time between 200 and 300 ms for optimal nulling of normal myocardium. The presence of LGE was qualitatively evaluated by a clinical expert (Level II or III certified [21]) blinded to all strain results, but with access to patients’ clinical data, to identify areas of hyper-enhancement in the myocardium consistent with either post-infarct scar or cardiac involvement in infiltrative disease [22].

### Reproducibility analysis

In a subset of 10 randomly selected patients, measurements were repeated by the same observer, two weeks after the first analysis (to prevent recall bias) and by a second independent observer for every modality and technique, all blinded to prior measurements and LGE findings.

### Statistical analysis

All values were assessed for normality using the Shapiro-Wilk test. Normally distributed data is expressed as mean ± SD, non-normally distributed data using median and interquartile range (IQR). Linear regression and Bland-Altman analyses were used to determine inter-technique agreement between STE, FT and SENC for GLS and between FT and SENC for GCS. Intra- and inter-observer variability was expressed in terms of intraclass-correlations (ICC) and coefficients of variation (CoV). Receiver operating characteristics (ROC) - curves were generated to establish the relationship of each strain parameter (STE-GLS, FT-GLS, FT-GCS, SENC-GLS, SENC-GCS) to the presence of LGE and the area under curve (AUC) was calculated. A Mann-Whitney test was conducted to determine if strain values measured using each technique, differed significantly between the patient groups with and without LGE. Binary logistic regression analyses were performed to determine the associations between strain measurements and the presence of LGE, which was expressed in terms of odds ratios (OR). A *p*-value of ≤0.05 was considered significant in two-tailed tests. Variables that were significantly associated with LGE presence were checked for collinearity by Spearman rank correlations and entered into separate multivariate logistic regression models for each technique to avoid overfitting and identify strain parameters that were independently associated with LGE. Statistical analyses were conducted using SPSS (Version 25.0, Statistical Package for the Social Sciences (SSPS), International Business Machines, Inc., Armonk, New York, USA).

## Results

The patient cohort included 15 patients with ischemic heart disease, 33 patients with non-ischemic heart disease and two patients with clinical indications for CMR but no cardiac diagnosis. The average time between echocardiogram and CMR-exam was 8.5 ± 9.8 days, with 35 patients (68.6%) scanned within the same week, and the remaining 15 patients with 30 days. Strain was evaluable using all techniques in 44/50 (88%) patients. In four patients, echocardiographic GLS could not be measured for technical reasons related to image transfer, in one patient FT-GLS and in another patient SENC-GLS could not be determined due to insufficient image quality. Table 1 shows the baseline characteristics, as well as CMR and echocardiographic measurements. Analysis time ranged from 3 to 5 min for STE, 8–44 min for FT (majority of time needed for GLS measurement), and 4–7 min for SENC. Both GLS and GCS values were similar among the techniques used.

**Table 1:** Baseline Characteristics of the Patient Population (*n* = 50)

Age (years)	51 ± 9
Female, n (%)	26 (52%)
Median (IQR) BSA (m^2^)	1.91 (1.71–2.06)
Ischemic heart disease, n (%)	15 (30%)
Non-ischemic heart disease, n (%)	33 (66%)
No cardiac diagnosis, n (%)	2 (4%)
LVEF (from CMR) (%)	56 (38–61)
Median (IQR) LVEDV Index (ml/m^2^)	87 (69–113)
Median (IQR) LVESV Index (ml/m^2^)	37 (30–56)
Average LV Mass Index (g/m^2^)	61.16 ± 24.95
LGE present, n (%)	22 (44%)
Median (IQR) GLS for Echo (*n* = 46)	−15.8 (−18.9 to −12.1)
Median (IQR) GLS for FT (n = 50)	−15.4 (− 18.4 to −10.6)
Median (IQR) GLS for SENC (n = 50)	− 14.9 (− 19.3 to − 11.1)
Median (IQR) GCS for FT (n = 50)	−14.3 (− 18.3 to − 11.1)
Median (IQR) GCS for SENC (n = 50)	− 13.7 (− 15.5 to − 10.8)

Abbreviations: *BSA* Body surface area, *LVEF* left ventricular ejection fraction, *LVEDV*, *LVESV* left ventricular end diastolic/end systolic volume, *LGE* late gadolinium enhancement, *GLS* global longitudinal strain

**Fig. 5:**
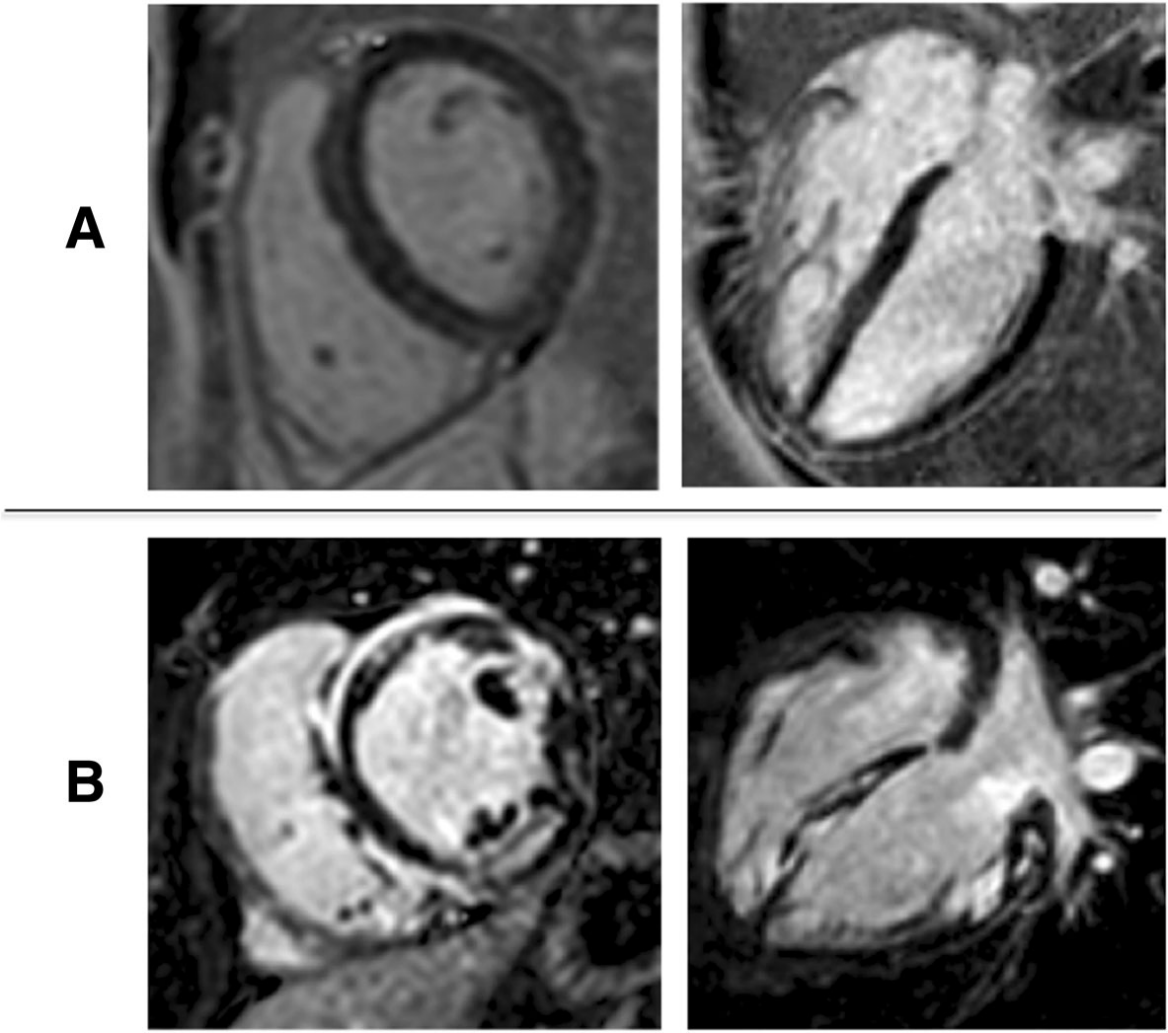
Example of LGE (short axis, 4Ch) in the same two patients as in Fig. 2. The ventricle of the patient (**a**) appears uniformly unenhanced, whereas in the sarcoidosis patient (**b**), there is diffuse, patchy enhancement in most myocardial segments

Figures 2, 3, 4 and 5 show examples of STE, FT, SENC and LGE analyses of two patients: one patient with no cardiac diagnosis (Figs. 2a, 3a, 4a and 5a) and another with a cardiac manifestation of sarcoidosis (Figs. 2b, 3b, 4b and 5b). Compared to the former case (Figs. 2a, 3a and 4a), the sarcoidosis patient had a peak GLS/GCS magnitude that was lower, as depicted by the strain curves obtained by all 3 imaging modalities/techniques (Figs. 2b, 3b and 4b). Figure 5 shows the corresponding LGE images of these two patients, depicting diffuse, patchy enhancement in most myocardial segments in the sarcoidosis patient **(**Fig. 5b), while the ventricle of the other patient (Fig. 5a) appears uniformly unenhanced.

### Inter-technique agreement

Table 2 summarizes the results of inter-technique agreement, including linear regression and Bland-Altman analysis. GLS measurements showed high levels of inter-modality agreement, reflected by good correlations: r-values of 0.71 and 0.75 between STE and FT and SENC, respectively. The biases were small (all < 1% in strain units, corresponding to approximately 5% of the mean measured value; NS) with limits of agreement of − 7 to 8% in strain units (corresponding to approximately 30–40% of the mean measured value), reflecting good inter-technique agreement but possible discordance in some individual patients. The agreement between the CMR techniques (FT and SENC) was better for GLS than GCS (r = 0.81 vs 0.67) and a smaller bias (− 0.2 vs 1.0%).

**Table 2:** Results of the linear- regression and Bland-Altman analyses to determine inter-technique agreement between the different modalities and techniques

	r	p	Bias (%)	LOA (%)	p
LV-GLS					
Echo vs. FT	0.71	< 0.001	0.9	−5.8 to 7.6	0.07
Echo vs. SENC	0.75	< 0.001	0.6	−5.9 to 7.2	0.21
FT vs. SENC	0.81	< 0.001	−0.2	−6.6 to 6.3	0.72
LV-GCS					
FT vs. SENC	0.67	< 0.001	1.0	−5.8 to 7.8	0.05

### Reproducibility analysis

Intra- and inter-observer variability, for both GLS and GCS, was very low for all modalities and techniques tested, reflecting excellent reliability based on ICC values of 0.86–0.99 and CoV 3–13% (Table 3**)**. Regarding both modalities and techniques, intra- and inter-observer variability for GLS measurements were the lowest for SENC, represented by the highest ICC and the lowest CoV values. Concerning CMR-derived GCS, the intra-observer variability between FT and SENC was similar, while the inter-observer variability was lower for FT.

**Table 3:** Results of the Reproducibility Analysis

			ICC	CoV
Intra-Observer Variability	GLS	STE	0.94	0.07 ± 0.05
		FT	0.89	0.13 ± 0.12
		SENC	0.99	0.04 ± 0.02
	GCS	FT	0.98	0.03 ± 0.03
		SENC	0.98	0.05 ± 0.05
Inter-Observer Variability	GLS	STE	0.91	0.07 ± 0.08
		FT	0.86	0.11 ± 0.19
		SENC	0.99	0.03 ± 0.03
	GCS	FT	0.99	0.03 ± 0.03
		SENC	0.94	0.08 ± 0.04

### Association between strain and LGE

LGE was present in 22 (44%) patients. In 9/22 (41%) patients, LGE pattern was consistent with prior myocardial infarction, in 13/22 (59%) patients, LGE pattern suggested an underlying fibrosing, infiltrative (e.g. cardiac amyloidosis) or inflammatory process (e.g. myocarditis), recognized by the location (not matching with vessel territories) and pattern (diffuse, patchy) of LGE.

**Fig. 6:**
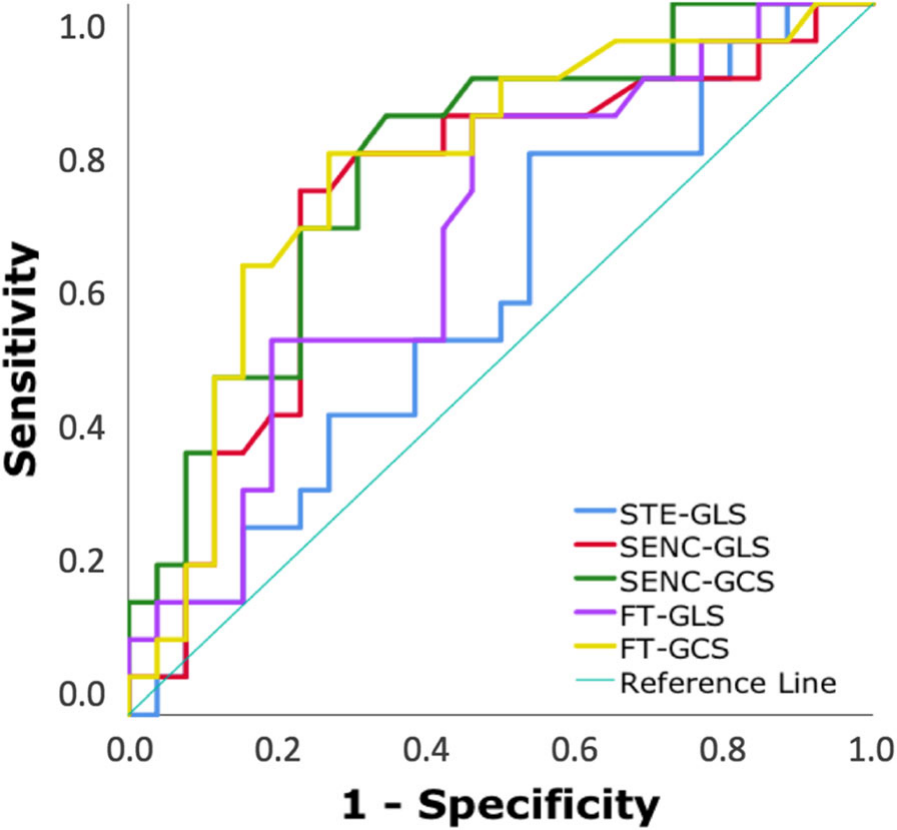
Receiver operating characteristic (ROC)- curves depicting the relationship between strain parameters and the presence of LGE

Table 4 and Fig. 6 summarize median strain values of patients with and without LGE and the results of the Mann-Whitney test. Table 5 displays the results of the ROC-analysis and the logistic regression. There was a significant difference in strain between patients with and without LGE when measured using FT or SENC, but not with STE. Both SENC- and FT-derived GLS and GCS were significantly associated with LGE, while STE GLS was not. Irrespective of CMR-technique, the association with LGE was stronger for GCS than for GLS and STE GLS. Interestingly, CMR-derived GCS showed higher odds ratios than GLS, with SENC-derived GCS having the highest OR value. The two separate multivariate logistic regression models accounting for GLS and GCS derived by FT and SENC **(**Table 6**)** showed that GCS from either technique was a significant independent factor associated with presence of LGE over GLS.

**Table 4:** Median strain values, measured using each technique, in patients without and with LGE and results of the Mann-Whitney test

	Patients without LGE	Patient group with LGE	p
Echo - GLS	−16.0 (−19.1 to −12.7)	−15.6 (−17.9 to −11.6)	0.212
FT - GLS	−17.3 (−18.8 to − 13.1)	− 12.5 (15.7 to −10.0)	0.003
FT - GCS	− 17.8 (− 19.1 to − 13.5)	− 12.0 (−14.3 to − 9.10)	< 0.001
SENC - GLS	−17.3 (− 20.2 to − 14.0)	− 13.5 (− 14.9 to − 10.2)	0.010
SENC - GCS	−14.8 (− 17.5 to − 12.9)	− 12.4 (− 13.8 to − 8.90)	< 0.001

Data presented as median (interquartile range)

**Table 5:** Results of ROC analysis and the univariate logistic regression analysis, demonstrating the association between strain measurements and LGE

	ROC analysis			Univariate logistic regression		
	AUC	95% CI	p	OR	95% CI	p
Echo - GLS	0.58	0.42–0.75	0.346	1.09	0.95–1.24	0.213
FT - GLS	0.67	0.52–0.83	0.048	1.19	1.04–1.36	0.013
FT - GCS	0.77	0.62–0.91	0.003	1.30	1.11–1.53	0.001
SENC - GLS	0.72	0.57–0.88	0.011	1.18	1.04–1.34	0.010
SENC - GCS	0.78	0.64–0.91	0.002	1.41	1.14–1.74	0.002

**Table 6:** Results of the multivariate logistic regression analysis for FT (Model 1)- and SENC (Model 2)-derived GLS and GCS, showing the strength of the association between strain measurements and LGE

	OR	95% CI	p
Model 1			
FT - GLS	0.95	0.75–1.22	0.696
FT - GCS	1.35	1.01–1.81	0.041
Model 2			
SENC - GLS	1.02	0.86–1.22	0.828
SENC - GCS	1.38	1.04–1.82	0.025

## Discussion

Echocardiography is typically the first imaging modality used to assess LV function in clinical routine, due to its low cost and widespread availability. In recent years, GLS using STE has been increasingly considered as an important parameter beyond the conventional measure of EF. An important step to make STE-derived GLS part of the clinical routine was the recent publication of a consensus document to standardize strain measurements [20]. Although several CMR-based strain measurement techniques have been described, this methodology has yet to be widely adopted into the clinical realm, and there is no consensus on what the optimal approach is. Similar to echocardiographic strain measurements, CMR strain also needs standardization prior to widespread clinical use.

Our study was designed as a step in this direction by comparing strain measurements obtained using the different modalities and techniques. In our study group: 1) on the average, there was a good inter-modality agreement between GLS from STE and CMR (both FT and SENC) and the agreement between CMR techniques was better for GLS than GCS; 2) in all comparisons, the limits of agreement were rather wide, indicating possible discordance in individual patients, 3) strain analyses, irrespective of modality or technique, were highly reproducible; and 4) STE-GLS was not significantly associated with LGE, whereas CMR-derived strain was, and the relationship was stronger for GCS than for GLS.

The good inter-modality agreement between GLS measured using CMR- and STE-derived strain is in line with most previous studies [23–26], although there is no consensus on this, as poor correlations have also been described [27]. Of note, previous authors have reported limits of agreement for both modalities and techniques that were similar or wider than in our study, indicating that inter-technique differences may be considerable in individual patients [23, 25, 26]. We also observed inter-technique agreement being higher for GLS than for GCS. One factor that may be affecting inter-modality and inter-technique agreement is the variability in strain measurements between vendors, even when using the same technique [28–31]. Also, intrinsic methodological differences may be an additional contributing factor. Importantly, however, the low inter- and intra-observer variability found in our study for both modalities and techniques, is similar to previous studies [17, 23, 26, 27, 32, 33], confirming that myocardial strain derived by both STE and CMR-techniques is highly reproducible. From a clinical perspective, the high reproducibility implies that strain techniques may be particularly valuable in the follow-up of the course of a patient’s disease. Nevertheless, it would be important to adhere to the same technique and to refrain from using different modalities interchangeably. Although no variation in clinical characteristics of the patients were noted between the two exams, we could not rule out changes in strain related to different loading conditions during the CMR and echocardiographic imaging, which were not performed on the same day.

In our study group, CMR-derived strain was significantly and independently associated with LGE, whereas STE-GLS was not. This relationship was stronger for GCS than for GLS. To our knowledge, this is the first study to establish the association between strain parameters and LGE for more than two modalities or techniques in the same patient cohort. Although echocardiography and CMR examinations were not performed on the same day, it is unlikely that differences in loading conditions alone could explain why CMR-derived strain was more closely associated with presence of scar than echocardiographic strain, because scar is not load dependent and was most likely consistently present during both examinations.

Moreover, our study group was composed of patients with heart disease of ischemic and non-ischemic etiology, unlike previous studies that focused on one specific diagnosis. Siegel et al. compared STE and FT in patients with Duchenne muscular dystrophy, and reported that, similar to our observation, FT was able to demonstrate presence of LGE, whereas STE was not [33]. However, their study used a small number of patients with a rare diagnosis. Altiok et al. only investigated GCS by STE and SENC in a group of patients with ischemic heart disease and concluded that both modalities were significantly associated with LGE [12]. Several previous studies of STE-GLS reported an association with LGE [11, 34, 35], which is contrary to our findings. This is probably due to the differences in patient populations (e.g. percentage of patients with LGE of ischemic origin) and the potential underlying differences in the prevalence, severity and specific patterns of strain abnormalities, as well as differences in measurement methodologies used.

When comparing the relationship with LGE for the CMR-techniques, we found that they performed similarly and that the relationship with LGE was stronger for GCS than for GLS, irrespective of the technique. Previous studies that investigated the association of CMR-derived strain with LGE in patients with acute ischemic heart disease also reported superior association for GCS compared to GLS when using both SENC [1, 17] and FT [14]. Up to this point, only a limited number of studies with small patient cohorts focused on either non-ischemic heart diseases [36–38] or chronic infarctions [17, 39]. Further studies are needed to investigate the differences in strain behavior and the association between strain and LGE in different patient populations.

### Clinical implications

Our results show that abnormal GCS measured by CMR-derived techniques indicates that LGE may be present. This may be clinically important when contrast use is problematic. Therefore, CMR may be useful in patients with suspected scar or other forms of myocardial damage, even when LGE imaging cannot be performed. Additionally, larger prospective studies to validate our results, and standardization of CMR-derived strain techniques are needed in order to facilitate the integration of this approach into clinical routine.

### Limitations

This is a retrospective study with the intrinsic limitations of no fully standardized protocol of image acquisition, resulting in variable image quality that may have affected strain measurements. Also, this was a single-center study with a relatively small sample size, which requires further validation for the conclusions to be generalized. One of the consequences of the small sample size was that it did not allow us to include more than two variables in the multivariate logistic regression analysis. Also, it would be interesting to compare patients with ischemic heart disease (focal LGE) and those with non-ischemic etiology (diffuse LGE). However, our retrospective study was designed to include all patients who underwent CMR with SENC and echocardiography (within a month of each other) over a 1-year period, resulting in a total of 50 studies suitable for analysis. Unfortunately, the number of patients in each of the above two categories was too small to allow statistically meaningful comparisons.

Because segmental strain is not a commonly used parameter due to inferior reproducibility and inter-technique agreement compared to global strain [40, 41], we focused only on global strain, which does not provide information on the location of scar. Moreover, this aspect of our study was motivated by the fact that our study included comparisons between two different CMR techniques used in the same setting and same imaging planes against echocardiography, which is a completely independent imaging modality. Because spatial registration between modalities is a well-known problem, we felt that segmental comparisons would be unfairly stacked against echocardiography because potential segment misalignment would affect these comparisons more than comparisons of global strain. Furthermore, intra-modality comparisons between SENC and FT would not be feasible in all patients because images of the scar may not have been acquired in both the long- and short-axis planes, which are utilized differently by the two CMR techniques. Nevertheless, it would be valuable to conduct analysis of regional strain in order to determine whether strain can reliably detect the location of scar, rather than its presence alone. In addition, the relationship between quantitative burden of LGE and strain was outside the scope of our study, which was designed to primarily investigate the inter-technique agreement and reproducibility of strain measurements.

Additionally, we did not include CMR-tagging in our study, although it is the current reference standard for strain measurements. Our study did not include tagging because various studies have already reported FT and SENC to be accurate compared to this reference [17, 42, 43], although the FT algorithm we used has not yet been evaluated on a large scale and may differ from the algorithms implemented in other software packages. We also did not investigate displacement encoding with stimulated echo (DENSE) strain imaging, although it would be interesting to include this modality as well, because there is no consensus in the literature regarding the level of inter-technique agreement between FT, SENC, and DENSE [41, 42].

## Conclusion

We found good inter-technique agreement in strain measurements between STE and CMR techniques and among CMR techniques, with small biases but considerable limits of agreement, indicating possible discordance in individual patients. Importantly, all strain measurements were highly reproducible, irrespective of modality or analysis technique. When measured by any technique, CMR-GCS was the strain parameter most related with LGE in our cohort and may potentially be considered as a surrogate for LGE when contrast administration is contraindicated.

## Data Availability

Data supporting the results reported in the manuscript can be found in a computer workstation in the Cardiac Imaging Laboratory at the University of Chicago.

## Abbreviations

AHA: American Heart Association
AUC: Area under the curve
CMR: Cardiovascular magnetic resonance
DENSE: Displacement encoding with stimulated echoes
ED: End-diastole
EF: Ejection fraction
ES: End-systole
FT: Feature tracking
GCS: Global circumferential strain
GLS: Global longitudinal strain
LGE: Late gadolinium enhancement
LV: Left ventricle/left ventricular
OR: Odds ratio
ROC: Receiver operator curve
SENC: Strain encoding
STE: Speckle tracking echocardiography
